# Simple and High Yield Synthesis of Metal-Polymer Nanocomposites: The Role of Theta-Centrifugation as an Essential Purification Step

**DOI:** 10.3390/polym9120659

**Published:** 2017-11-30

**Authors:** Patrick Hummel, Arne Lerch, Sebastian Manfred Goller, Matthias Karg, Markus Retsch

**Affiliations:** 1 Department of Chemistry, University of Bayreuth, Universitätsstr. 30, 95447 Bayreuth, Germany; patrick.hummel@uni-bayreuth.de (P.H.); sebastian.goller@uni-bayreuth.de (S.M.G.); 2 Physical Chemistry I, Heinrich-Heine-University Düsseldorf, Universitätsstr. 1, 40204 Düsseldorf, Germany; Arne.Lerch@hhu.de (A.L.); matthias.karg@hhu.de (M.K.)

**Keywords:** steric stabilization, nanoparticles, polymer ligand, ligand exchange

## Abstract

Nanocomposites are an important materials class, which strives to foster synergistic effects from the intimate mixture of two vastly different materials. Inorganic nanoparticles decorated with polymer ligands, for instance, aim to combine the processing flexibility of polymers with the mechanical robustness of solid state materials. The fabrication and purification of such composite nanoparticles, however, still presents a synthetic challenge. Here, we present a simple synthesis of silver polystyrene nanocomposites with a controllable interparticle distance. The interparticle distance can be well-controlled with a few nanometer precision using polystyrene ligands with various molecular weights. The nanoparticle and polymer ligand synthesis yield both materials on gram scales. Consequently, the polymer nanocomposites can also be fabricated in such large amounts. Most importantly, we introduce Θ-centrifugation as a purification method, which is capable of purifying large nanocomposite batches in a reproducible manner. We employ a range of characterization methods to prove the successful purification procedure, such as transmission electron microscopy, thermogravimetric analysis, and dynamic light scattering. Our contribution will be of high interest for many groups working on nanocomposite materials, where the sample purification has been a challenge up to now.

## 1. Introduction

Polymer nanocomposites (PNC) are a material class of high interest. Superior material properties can be achieved by the synergistic combination of the soft polymer coating and the rigid inorganic core. Depending on the combination of polymer and core material a range of properties can be varied. As an example, the incorporation of nanoclay sheets reinforces the mechanical properties, improves barrier properties and flame retardation [1]. PNCs filled with semiconductor nanocrystals have the potential for light conversion applications [2], such as light emitting diodes [3,4]. In addition to inorganic fillers, also organic fillers like graphene [5,6,7,8], graphene-oxide [9], (modified) carbon fibers [10] and multi-walled carbon nanotubes (MWCNT) [11,12], can be used and increase the multifunctionality of PNCs further. Due to the improved mechanical, thermal, and electrical properties they show potential in many applications. For example, graphene-filled thermoplastic elastomers can be used as piezoresistive sensors [5], strain sensors [6,8], and organic vapor sensors [7].

PNCs can be also produced by sputter deposition of metals on a polymer film. The deposition rate controls the cluster growth and therefore the size, shape and morphology of the metal clusters. Hence, the structure and consequently the effective properties can be tailored in a very controlled manner by the variation of the deposition rate [13,14,15]. However, only 2D arrangements are possible with this method. In this paper we focus on systems with the potential to form 3D arrangements of PNCs. A paramount precondition for the fabrication of high-performance polymer nanocomposites is the strict exclusion of nanoparticle aggregation or demixing with the polymer matrix [16]. Therefore, it is of great importance to ensure a good stability of the nanoparticles. A conventional method is to coat the nanoparticles with the same polymer as the matrix. Polymer brushes are often used for this purpose.

There exist two main approaches to form a polymer brush around a nanoparticle, grafting-from and grafting-to. In the grafting-from approach molecules, like initiators for atomic transfer radical polymerization (ATRP) [17] or chain transfer agents (CTAs) for reversible addition-fragmentation chain transfer (RAFT) polymerizations [18], are immobilized on the particle surface. The drawbacks of this method are the development of new initiator coupling reactions. Furthermore, the particles have to be colloidally stable under the polymerization conditions. The grafting-to method is more flexible with respect to polymerization conditions because the polymers are synthesized separately. Afterwards they are attached to the particle surface by a chemical reaction or physisorption. One drawback of grafting-to is the usually lower grafting density, due to the steric repulsion of the coiled polymer chains during attachment to the particle surface. A common grafting-to approach is the ligand exchange method. Here, the surfactants used during nanoparticle synthesis are exchanged by functional polymer ligands. Already a wide range of particles, functional groups and polymers was demonstrated by this method [19,20,21,22]. The biggest advantage is the complete segregation of nanoparticle and polymer synthesis. Thus, on the one hand, high yield nanoparticle synthesis routes can be exploited. On the other hand, the polymerization technique can be freely chosen. Here RAFT synthesis has excelled due to a large variety of monomers and easy functionalization [23,24]. The CTA can be readily used as functional group itself [24], a functionalized CTA can be used or the polymer chains can be post-functionalized [25]. All grafting-to methods suffer from the drawback that a rigorous workup is necessary to remove the excess of free ligand from the functionalized nanocomposite particle. This is typically achieved by selective precipitation and centrifugation. The selective precipitation is a challenging task and needs high experimental skills.

In this work we present a polymer nanocomposite synthesis based on silver cores stabilized by thiol functionalized polystyrene ligands. We combine well known and simple synthesis routes with access to high yields of silver nanoparticles and end-group functionalized polystyrene. Furthermore, we use a ligand exchange process to obtain densely grafted silver polystyrene nanocomposites and demonstrate a new alternative, and in our opinion superior, purification route based on Θ-condition centrifugation. Finally, we demonstrate a high control of the edge-to-edge particle distance in our polymer nanocomposites, due to the systematic dependency of the interparticle distance on the molecular weight of the ligands. This tailoring of the interparticle distance is a prerequisite for applications, like efficient surface-enhanced Raman scattering (SERS) sensors [26].

## 2. Materials and Methods

### 2.1. Materials

All solvents used were pro analysi (p.a.) grade. Prior to polymerization the inhibitor of the styrene (Sigma, St. Louis, MO, USA, ≥99%) was removed by an aluminum oxide column. 2-cyano-propyl dodecyl trithiocarbonate (CPDTTC) (Sigma, St. Louis, MO, USA, 97%), 1-butylamine (Alfa Aesar, Ward Hill, MA, USA, 99%), silver nitrate (Alfa Aesar, Ward Hill, MA, USA, 99.9995%), triethylamine (Alfa Aesar, Ward Hill, MA, USA, 99%), and sodium myristate (Sigma, St. Louis, MO, USA, ≥99%) were all used as received.

### 2.2. RAFT Synthesis

Our RAFT polymerization was conducted according to the work of Mayadunne et al. [27]. The desired ratio of 2-cyano-propyl dodecyl trithiocarbonate and styrene were weighed in a single-neck flask equipped with a stirrer bar. The flask was sealed with a septum and degassed with argon for 30 min. Afterwards, the reaction was started by heating the flask to 110 °C. The conversion was monitored by ^1^H-NMR measurements. When the desired conversion was reached, the reaction was stopped by cooling the flask in an ice bath. Then the reaction mixture was dissolved in tetrahydrofuran (THF) and separated from remaining styrene by precipitation in cold methanol. For the shortest polymer (1000 g/mol) a methanol:water 3:2 mixture was used. The reaction conditions of all polymerizations are summarized in Table 1.

For the aminolysis the polymers were dissolved in toluene and a 25-fold excess of butylamine was added. The solution was stirred for 24 h. Afterwards the solution was extracted three times with brine and precipitated in cold methanol.

### 2.3. Nanoparticle Synthesis

The nanoparticle synthesis was performed according to Yamamoto et al. [28]. The precursor for the silver nanoparticle synthesis, silver myristate, was received by mixing equal molarities of sodium myristate and silver nitrate. The white precipitate was filtered and dried overnight in a vacuum oven.

For a standard formulation of the nanoparticle synthesis 1 g silver myristate and 22 mL triethylamine were used. Additionally, reactions with up to 10 g silver myristate and 220 mL trimethylamine were performed. Both reactants were mixed in a Schlenk flask connected to a reflux condenser. The flask was then purged with argon for 30 min. Hereafter, the reaction mixture was heated to 80 °C and held at this temperature for 4 h. After around 15 min the white dispersion slowly becomes yellow and brown. Finally, a homogeneous black solution is formed.

The nanoparticles were purified by precipitation with acetone with subsequent centrifugation at 5095 relative centrifugal force (rcf) for 30 min. They were dissolved in toluene and the purification step was repeated two times.

### 2.4. Ligand Exchange

An excess of polymer ligand was used for the ligand exchange. The excess was calculated based on the amount of ligand needed for a potential grafting density of 10 nm^−2^. The required amount of polystyrene was dissolved in toluene and the corresponding nanoparticle dispersion was added dropwise. This mixture was stirred for 65 h.

In the next step, the nanocomposites were quantitatively precipitated with ethanol. The precipitate was separated by centrifugation at 5095 rcf for 30 min and redispersed in toluene. This procedure was repeated two times, but after the last centrifugation the precipitate was redispersed in cyclohexane instead of toluene. Subsequently the nanocomposites were centrifuged at 10,000–50,000 rcf at the Θ-temperature of styrene in cyclohexane, 34 °C. We want to mention that a centrifugal force of 10,000–15,000 rcf was already sufficient to sediment the nanocomposite particles. Higher centrifugation forces speed up the work up procedure. The centrifugations were usually proceeded overnight. We note that compatibility of centrifugal tubes with elevated temperatures and solvents should be tested prior to centrifugation overnight.

### 2.5. Characterization

^1^H-NMR measurements were recorded on a Bruker Avance 300 A (300.13 MHz, Billerica, MA, USA) spectrometer using CDCl_3_ as the solvent.

The molecular weight distributions were determined by size exclusion chromatography (SEC) with THF as eluent with a flow rate of 0.5 mL·min^−1^ at 25 °C, using a linear polystyrene standard obtained from Polymer Standard Service (PSS, Mainz, Germany, the Knauer, Berlin, Germany system is equipped with a PSS-SDV (10 µm) 50 mm × 8 mm column, two 600 mm × 8 mm columns, and a differential refractive index detector).

Ultraviolet-visible (UV-VIS) spectra were measured with an Analytik Jena SPECORD 250 Plus spectrophotometer (Jena, Germany). As cuvettes, high-precision cells made of quartz SUPRASIL from Hellma Analytics (Müllheim, Germany) with a light path of 10 mm were used.

A Sigma 3-30KS centrifuge (Sigma Laborzentrifugen GmbH, Osterode am Harz, Germany) equipped with a Sigma 19776-H or a Sigma 12111-H rotor was used for centrifugation procedures. The latter rotor was only used for the Θ-centrifugation with 10 mL Sigma 15,000 polyfluor tubes, whereas the first was used for both, normal and Θ-centrifugation steps with 50 mL polypropylene tubes purchased from VWR (Radnor, PA, USA). 

Light scattering experiments were performed on a 3D spectrometer from LS Instruments AG (Fribourg, Switzerland) equipped with a HeNe-laser (λ = 632.8 nm, maximum constant power output of 35 mW). Experiments were performed in 2D operation. The samples dispersed in cyclohexane were heated to 45 °C and directly filtered (hydrophobic PTFE filter, pore size 5 µm) into dust free quartz cuvettes with a diameter of 10 mm (Hellma Analytics, Müllheim, Germany). The quartz cuvettes were heated to 45 °C and vortexed for aggregates to disperse completely. For the measurements the cuvettes were placed in a temperature controlled decalin bath (stability ± 100 mK) preheated to a temperature of 44 °C. The temperature was followed by a PT100 thermoelement placed close to the sample position in the decalin bath. Measurements were perfomed after an equilibration time of 30 min at temperatures of 44 °C followed by 34 °C followed by 24 °C. The scattered light was detected by two avalanche-photo-diodes operating in pseudo cross correlation. Three intensity-time autocorrelation functions were measured for 30 s at a scattering angle of θ = 90 ° for all three temperatures. Analysis of the recorded autocorrelation functions was performed by CONTIN analysis (inverse Laplace transformation) with the software AfterALV (v1.06d, Dullware Inc., Amsterdam, The Netherlands).

Thermal gravimetric analysis (TGA) was performed on a Mettler Toledo TGA/STDA 851e Star System (Columbus, OH, USA) at a heating rate of 10 K·min^−1^ in a temperature range between 30 °C and 700 °C and a nitrogen flow of 50 mL·min^−1^.

A JEOL JEM-2200FS (Akishima, Japan) and a Zeiss EM922 Omega (Jena, Germany) transmission electron microscope (TEM) were used to record TEM images of the nanoparticle and nanocomposite samples. ImageJ 1.49v (National Institute of Mental Health, Bethesda, MD, USA) was utilized to evaluate the TEM images. This analysis provided the average particle size and the edge-to-edge interparticle distance.

## 3. Results and Discussion

**Figure 1:**
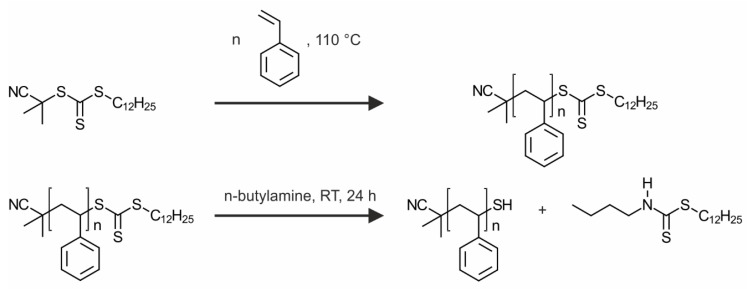
Reaction scheme of the RAFT polymerization (**top**) and the aminolysis to yield a thiol functionalization (**bottom**).

The polystyrene ligands were synthesized by a reversible addition-fragmentation chain transfer (RAFT) polymerization. We chose thermal initiation at 110 °C and used CPDTTC as the CTA. The polymerization was conducted in bulk. The reaction scheme is shown in Figure 1.

The desired molecular weight was adjusted by the ratio of styrene and CTA and the conversion of the reaction. For calculation of the molecular weight we used a simplified equation from the literature [27]:

(1) $$M_{n}\left(\mathrm{calc}\right)=\frac{\left[\mathrm{styrene}\right]}{\left[\mathrm{CTA}\right]}\cdot \mathrm{conversion}\cdot M_{\mathrm{styrene}},$$

**Figure 2:**
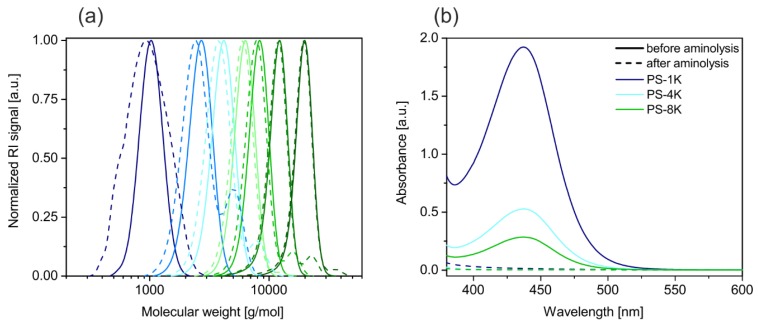
(**a**) Molecular weight distributions of the polystyrene ligands before (solid lines) and after (dashed lines) the aminolysis. (**b**) UV-VIS spectra of the 1 K, 4 K, and 8 K ligands before (solid lines) and after (dashed lines) the aminolysis. The absence of the n → π* transition proves the successful reaction. The absorbance decreases with increasing molecular weight, which correlates well to the ratio between trithiocarbonate end-groups and the ligand length.

The calculated number averages of the molecular weight (*M*_n_(calc)) are in good agreement with the experimental *M*_n_ values, as it can be seen in Table 1. Especially in the range from 1000 to 8000 g/mol the predictions were accurately met. Beyond 8000 g/mol the deviation increases slightly. Nevertheless, the high conformity of the calculated and experimental *M*_n_ and the low polydispersities of 1.05 show the high control over the reaction. The narrow distribution of the molecular weights before aminolysis is presented by the solid lines in Figure 2a.

Based on the RAFT polymerization, every chain bears a trithiocarbonate end-group. We converted this to a thiol group, which provides a smaller footprint anchor group for the silver nanoparticle surface. Consequently, higher grafting densities are likely to be reached. For the thiol generation, an aminolysis is conducted, as illustrated in Figure 1. This decreases the molecular weight slightly (dashed lines in Figure 2a) and in some samples a second peak at the double molecular weight emerges. The second peak belongs to a small fraction of chains, which have been connected by disulfide bonds. The presence of disulfide bonds evidences the successful cleavage of the trithiocarbonate groups. It is assumed that the relatively weak disulfide bonds do not disturb the ligand exchange, because they will dissociate and form stronger Ag–S bonds, as it was reported for gold interfaces [29]. Furthermore, the color of the polymers changed from yellow to white after the aminolysis. The UV-VIS spectra of three different ligands before and after the aminolysis are given in Figure 2b. The solid lines present the spectra before the aminolysis. A peak at around 420 nm, attributed to the n → π* transition can be seen [30]. This peak completely vanishes in the spectra after the aminolysis represented by the dashed lines. Overall, we could show that the RAFT polymerization is a straightforward method to yield thiol terminated polystyrene ligands with low polydispersity and a good control of the molecular weight. In addition, we want to note that RAFT synthesis is suitable for many other monomers. In consequence the synthesis could be easily assigned to design ligands from a wide range of polymers.

The synthesized polymers were then used in a homogeneous ligand exchange reaction. Therefore, an excess of polystyrene ligands was mixed with separately synthesized silver nanoparticles in toluene. The monodisperse silver nanoparticles were produced according to Yamamoto et al. [28]. The resulting particles are stabilized by myristate, which binds with its carboxylic moiety to the Ag nanoparticle surface. The thiol group of the polymer ligand replaces the myristate due to the weaker binding strength of the carboxylic acid groups. Still, a large excess of polymer ligand is needed to fully drive the exchange reaction towards completion [21]. The ligand exchange is additionally aided by a quantitative precipitation step. Here, the nanocomposite and the free polymer is completely precipitated by the addition of a non-solvent, in our case, ethanol. This step is crucial for the complete ligand exchange and the generation of a high grafting density, as the local polymer concentration at the nanoparticle surface is strongly increased by the precipitation. Subsequently, the sample is centrifuged and the precipitate with the nanocomposites and the free polymer is separated from the supernatant, which is enriched by the unbound myristate molecules [21]. After the complete functionalization of the Ag nanoparticle with the desired polymer ligand, the excess free polymer has to be removed. Several strategies can be followed for this. Ehlert et al. [21] introduced a second, so called selective, precipitation step. The non-solvent is added stepwise, until only the nancomposites precipitate, but not the free polymer. Experimentally, this is a great effort because the sample has to be centrifuged after every addition of non-solvent to check if there is still nanocomposite in the dispersion. Additionally, the added amount can be too high and an additional quantitative precipitation instead of a selective precipitation was performed. Dialysis represents a well-established alternative, but is mainly applicable to water-based polymers. Furthermore, dialysis is a time-consuming procedure and requires a large hydrodynamic size mismatch between the functionalized nanoparticle and the ligand.

**Figure 3:**
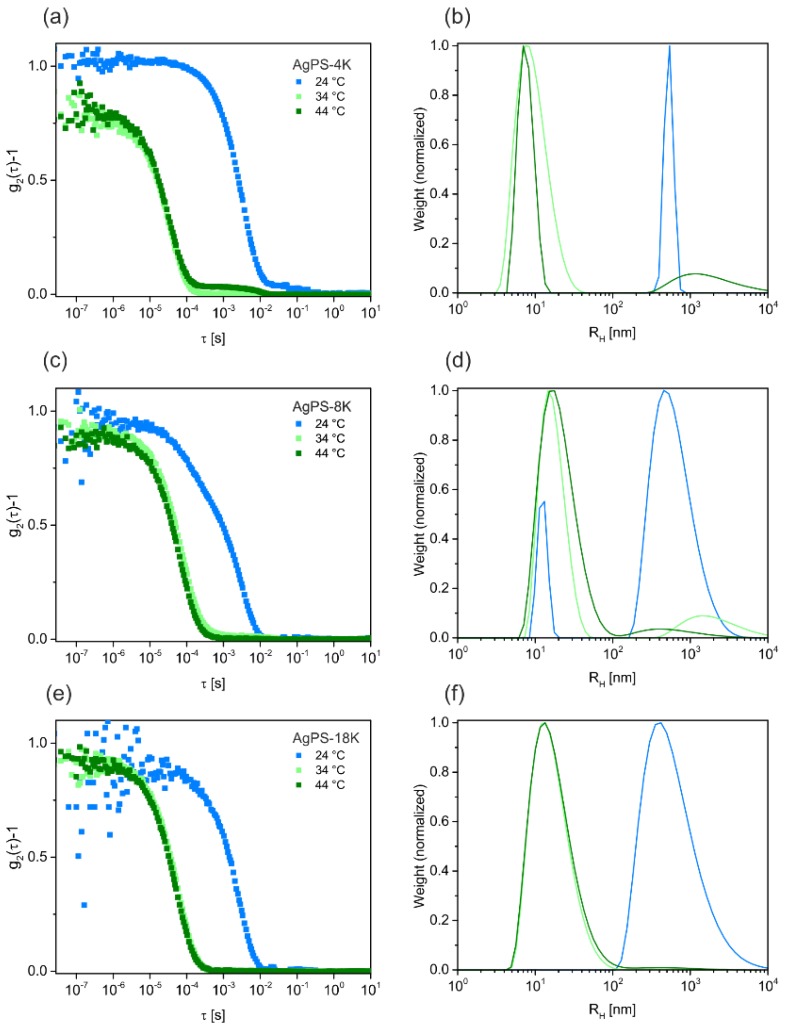
Dynamic light scattering measurements of PNCs in cyclohexane. Left panel: intensity-time autocorrelation functions. Right panel: corresponding size distribution of the samples obtained from CONTIN analysis. The measurements were performed at three different temperatures. Below (24 °C), at (34 °C) and above (44 °C) the Θ-temperature. The ligand length increases from top to botton: (**a**,**b**) AgPS-4K sample, (**c**,**d**) AgPS-8K sample and (**e**,**f**) AgPS18K sample. All samples show aggregates below the Θ-temperature.

For these reasons, we suggest another method to purify the nanocomposites in organic solvents from excess ligands. Instead of using solvent and non-solvent mixtures, we recommend to use a Θ-solvent and centrifugation at the Θ-temperature. This is a convenient and reproducible strategy, because one does not have to exactly adjust the right amount of non-solvent, but can set the temperature to Θ-conditions, which is well known for many polymer/solvent combinations. By definition, the enthalpic interaction between polymer and solvent is equal to the polymer-polymer interaction at the Θ-temperature. Consequently, the steric repulsion of the polymer brushes decreases [31]. Below the Θ-temperature the solvent becomes enthalpically unfavorable, while it approaches good solvent conditions at higher temperatures. This general behavior is known from the Flory-Huggins theory. Temperature-dependent dynamic light scattering (DLS) measurements on our nanocomposite particles confirm this behavior (Figure 3). Figure 3a,c,e show intensity-time autocorrelation functions measured for the PNCs with different molecular weight ligands at 24, 34 and 44 °C. At first glance one recognizes a monomodal decay at relatively short decay times for all samples measured at elevated temperatures (34 and 44 °C). Furthermore, the autocorrelation functions for each sample at these temperatures almost match, indicating only slight differences in object size. Only for the shortest polymer ligand (AgPS-4K) a small second decay is observed at longer decay times pointing to a small fraction of larger objects, i.e., aggregates. At 24 °C, i.e., well below the Θ-temperature, the decay of the autocorrelation functions is shifted significantly to longer decay times, which points to much larger objects. Figure 3b,d,f show distribution functions of the hydrodynamic radii *R*_h_ obtained from CONTIN analysis of the autocorrelation functions. These distribution profiles confirm the trends observed from the autocorrelation functions. The data show, that the PNCs form aggregates with large *R*_h_ values on the order of 500 nm below the Θ-temperature at 24 °C. In contrast, the PNCs remain dispersed at the Θ-temperature and above (34 and 44 °C, respectively). Only for the smaller samples, AgPS-4K and AgPS-8K, a small fraction of larger objects is found. We want to highlight that the latter size populations that we attribute to small fractions of aggregates resemble the minority of the samples since the data are intensity weighted and not number weighted. The DLS measurement confirms the expected influence of the temperature on the PNCs stability in solution.

**Figure 4:**
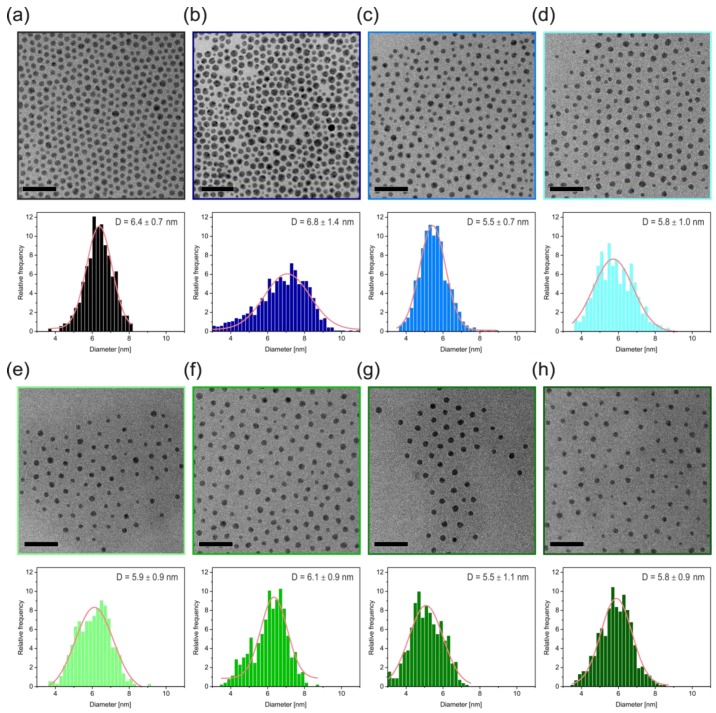
(**a**) Pure Ag@myristate nanoparticles. (**b**–**h**) nanocomposite samples with the following molecular weight of the polystyrene ligands: (**b**) 1 kg/mol; (**c**) 3 kg/mol; (**d**) 4 kg/mol; (**e**) 6 kg/mol; (**f**) 8 kg/mol; (**g**) 12 kg/mol; and (**h**) 18 kg/mol. The interparticle distance increases with increasing molecular weight. All scale bars are 50 nm. The histograms in the bottom panel show the size distributions of the Ag core diameter of the respective sample.

The Θ-temperature itself is a function of the polymer molecular weight. One can, therefore, expect that the grafted polymer chains possess a lower Θ-temperature than their freely dissolved counterparts. Distinct differences between the tethered and the free polymer additionally help the selective purification. At the Θ-temperature the tethered and free polymer will be less extended, compared to a good solvent [32]. Furthermore, the height contraction of tethered polymers is larger than the decrease of *R*_g_ of free polymer coils [33]. Consequently, the solubility of tethered polymer chains is much more decreased compared to the solubility of free polymer chains at Θ-conditions. Whereas the mentioned studies were performed for polymer brushes attached to planar surfaces, an analogous behavior can be expected for spherical brush particles. Hence, the steric stabilization is sufficiently suppressed and the polymer functionalized nanoparticles can be separated via centrifugation, whereas the free polymer chains remain dissolved in the supernatant. We applied this simplified purification method to a large range of polystyrene ligands with molecular weights ranging from 1 up to 18 kg/mol. The systematic increase in interparticle distance, observed in the TEM images in Figure 4, is a strong indication for the removal of all excess free polymer and the successful coating with the desired polymer ligand.

**Figure 5:**
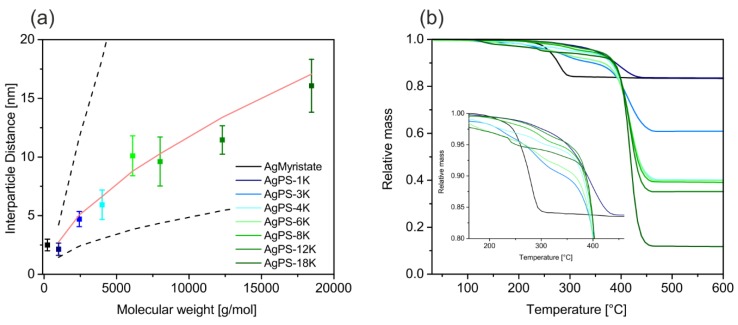
(**a**) The edge-to-edge interparticle distance in the nanocomposites increases systematically with higher molecular weight. The dashed lines present the upper (fully-stretched chain) and lower limit (random coil *R*_g_ ≈ *l*_0_N^1/2^). The red line was calculated by Equation (2) and represents the theoretical interparticle distance of a polymer brush in the semi dilute regime. (**b**) TGA curves of the nanocomposites and of pure silver particles with myristate ligand as a reference. The inset emphasizes the onset of thermal degradation. The myristate ligand decomposes at much lower temperatures than the polystyrene ligand.

The TEM images show monodisperse and well-separated particles. The monodispersity of the Ag core is pointed out by the size distribution histogram below the respective TEM image. All samples show a normal size distribution, as indicated by a Gaussian fit to the histogram. From this we obtained a mean diameter and standard deviation. Only the nanocomposite sample with the smallest ligand (AgPS-1K) shows a slightly increased size distribution. We suppose that some Ostwald ripening took place during the ligand exchange, owing to the reduced steric stabilization compared to longer ligands. In Figure 4a the pure Ag@myristate particles are presented. After the ligand exchange the myristate is substituted by polystyrene ligands. This directly translates into a molecular weight dependence of the interparticle distance (Figure 5). The interparticle distance, which we determined between the respective particle surfaces (i.e., edge-to-edge), is plotted against the molecular weight in Figure 5a. The interparticle distance ranges from around 2 to 16 nm and increases monotonically with the molecular weight. This allows a very controlled adjustment of the interparticle distance in such nanocomposite materials. The shortest polystyrene ligand with a *M*_n_ of 1000 g/mol features an even shorter interparticle distance as the myristate stabilized particles (2.5 nm), as already observed by Ehlert et al. [21]. This offers the opportunity not only to increase the interparticle distance by coating with polymer ligands, but also to decrease it.

The interplay between the degree of polymerization, the grafting density and the core particle curvature have been investigated by other groups in detail [34,35,36]. The theoretical brush height can be described by the Daoud-Cotton model for star polymers shown in Equation (2).

(2) $$h\propto l_{0}\cdot N^{\frac{3}{5}}\cdot f^{\frac{1}{5}}\cdot \nu^{\frac{1}{5}},$$

where l_0_ is the length of one monomer unit, *N* is the degree of polymerization, *f* the number of arms of the star-polymer and ν the second virial coefficient. It has been shown that their model can predict the brush height of polymer chains tethered to a particle surface, too. The dry interparticle distance of our grafted samples are well described by Equation (2), illustrated by the red line (Figure 5a). This can be assigned to the semi-dilute brush regime. The red line was calculated with the mean values of all PNC samples (*f* = 141). This is an unexpectedly low scaling behavior with respect to the ligand molecular weight and the high grafting density as determined below. We want to stress that the theory is based on brush stabilized nanoparticles in a solvent, while we investigated dried samples adsorbed on a TEM grid. Consequently, the particles on the TEM images are closely packed and possible entanglement between brushes of neighboring particles is not taken into account. Yet, the universal applicability of this scaling theory to all our samples with vastly different molecular weights corroborates the successful ligand exchange. Most importantly it rules out the presence of free, unbound polymer, which would result in larger and non-systematic interparticle distances.

We determined the grafting densities of the PNCs separately by TGA analysis. The grafting density *D* was calculated by Equation (3) [21]:

(3) $$D=\frac{4\cdot r_{n}^{3}\cdot \rho_{n}\cdot N_{A}\cdot \left(100-X_{n}\right)}{3\cdot {\left(2\cdot r_{n}\right)}^{2}\cdot M_{p}\cdot X_{n}},$$

where *r_n_* is the radius of the nanoparticle, ρ_n_ is the bulk density of silver, N_A_ the Avogadro constant, *M*_p_ the molecular weight of the ligand and *X*_n_ the weight percentage of the silver, measured by TGA. The TGA measurements of all nanocomposites are shown in Figure 5b. Additionally, a reference measurement of the pure Ag myristate particles before the ligand exchange is shown. It can be seen that the main step of thermal degradation shifts to higher temperatures when exchanging the myristate with the polystyrene ligands. No sign of residual myristate was found in the decomposition behavior of the polymer nanocomposites, indicating a quantitative ligand exchange. Furthermore, the residual mass depends on the molar mass of the polymeric ligand. The residual mass decreases with increasing ligand length.

The relatively low difference in residual Ag content in the Ag@PS-4K to 8K samples is ascribed to differences in the grafting densities in these samples. The grafting densities with the related silver contents and the interparticle distances are listed in Table 2. The grafting densities are 0.8 to 2.5 ligands per nm^2^. These are high grafting densities and compare to values reported for the ligand exchange method (1.2 ligands per nm^2^) [21]. Furthermore, our grafting densities are as high as the grafting density of CTA reported for grafting-from approaches by RAFT polymerization [37]. Accordingly, our particles show a higher polymer grafting density, because not every CTA will lead to a polymer chain in a grafting-from approach. Typically, the resulting grafting density in grafting-from approaches lies in the region of 0.6 ligands per nm^2^ [37,38,39]. Therefore, our homogeneous ligand exchange, in combination with quantitative precipitation and Θ-condition purification, results in highly-functionalized nanocomposites and even compares well to grafting-from approaches.

## 4. Conclusions

In this work we demonstrated a simple and high yield synthesis for nanocomposites composed of polymer functionalized silver nanoparticles. The 5 nm diameter silver cores are densely decorated by thiol-terminated polystyrene ligands, obtained from RAFT syntheses. The molecular weight was controlled by the ratio of styrene and chain transfer agent. Seven ligands of different lengths, determined by the molecular weight, were used in a ligand exchange process. We introduced the Θ-centrifugation as a new method to purify the polymer nanocomposites from excess polymer after the ligand exchange. The concept of this purification method was supported by temperature-dependent DLS measurements to demonstrate the nanocomposite stability. Stable particles were found close to, and above, the Θ-temperature, whereas aggregated particles formed at lower temperatures. The feasibility of the purification procedure was verified by TGA and TEM measurements. The systematic dependence of the interparticle distance on the molecular weight was manifested by TEM images. At the same time, high grafting densities comparable to grafting-from methods could be realized. Overall, our contribution highlights the strength of the grafting-to method to access well-defined nanocomposites. The introduction of an alternative purification protocol adds increased reproducibility and scalability to the fabrication of future nanocomposite materials.

## Figures and Tables

**Table 1:** Summary of the reaction conditions of the different polystyrene ligands.

Sample name	[Styrene]/[CTA]	Conversion	Reaction time	*M*_n_	*M*_n_(calc)	PDI
		%	h	g/mol	g/mol	
PS-1K	12	80.4	68	996	1000	1.05
PS-3K	38	76.2	50	2560	3080	1.05
PS-4K	48	81.5	67	3940	4070	1.05
PS-6K	96	75.5	28.5	6030	7660	1.04
PS-8K	96	82.6	47	7940	8260	1.04
PS-12K	154	92.2	25	11,380	14,770	1.04
PS-18K	288	79.1	32	18,850	23,730	1.04

**Table 2:** Overview of the silver content, grafting density, and edge-to-edge interparticle distance (IPD with standard deviation σ_IPD_) of the nanocomposite samples.

Sample name	Ag content	Grafting density	IPD	σ_IPD_
	wt %	ligands/nm^2^	nm	nm
Ag@PS-1K	83.6	1.2	2.1	0.5
Ag@PS-3K	60.9	1.4	4.8	1.3
Ag@PS-4K	44.8	2.0	5.9	1.3
Ag@PS-6K	39.9	1.4	10.1	1.9
Ag@PS-8K	39.1	1.1	9.6	2.1
Ag@PS-12K	35.2	0.8	11.3	2.4
Ag@PS-18K	11.8	2.5	16.1	1.7
